# Peptide Drugs in Gastrointestinal Tumors: Integrating Targeting, Delivery, and Therapeutic Actions for Synergistic Strategies

**DOI:** 10.3390/biom16030456

**Published:** 2026-03-18

**Authors:** Qian Ouyang, Guifang Wu, Anyi Chen, Rui Zhang, Shuai Xiao, Dong Guo, Qi Zhang, Chaojun Yan, Xing-Zhen Chen, Jingfeng Tang, Hao Lyu

**Affiliations:** 1 National “111” Center for Cellular Regulation and Molecular Pharmaceutics, Hubei University of Technology, Wuhan 430068, China; 2 Cooperative Innovation Center of Industrial Fermentation (Ministry of Education & Hubei Province), Hubei Key Laboratory of Industrial Microbiology, Hubei University of Technology, Wuhan 430068, China; 3 Membrane Protein Disease Research Group, Department of Physiology, Faculty of Medicine and Dentistry, University of Alberta, Edmonton, AB T6G 2R3, Canada

**Keywords:** gastrointestinal malignant tumors, peptide drugs, targeted treatment, drug delivery, anti-tumor treatment

## Abstract

Gastrointestinal malignant tumors account for approximately one-third of global cancer-related deaths, primarily including colorectal, gastric, pancreatic ductal adenocarcinoma, and hepatocellular carcinomas. These tumors have a high incidence, are often asymptomatic, and are prone to metastasis and recurrence, posing a significant public health burden. Although traditional methods such as radiotherapy and chemotherapy can delay disease progression, their nonspecific effects often lead to severe side effects and drug resistance, resulting in limited efficacy. Therefore, developing novel treatment strategies with high target specificity and favorable biological safety is a critical scientific issue in this field. Peptide drugs offer advantages such as good biocompatibility, low immunogenicity, diverse structures, and ease of modification, collectively demonstrating unique potential for tumor treatment. They can not only achieve precise delivery by specifically recognizing tumor receptors but can also directly interfere with signal transduction, metabolism, and immune regulation, producing multi-target antitumor effects. This article systematically reviews the research progress of peptide drugs in gastrointestinal tumors, focusing on their molecular mechanisms, delivery modification strategies, and the latest applications. It also summarizes the challenges and future directions for clinical translation, providing a theoretical foundation and future perspectives for the precise treatment of gastrointestinal tumors and the design of new drugs.

## 1. Introduction

Since insulin was first used to treat diabetes in 1921, research on peptide drugs has gradually become a significant focus in biomedicine [1,2]. Over the past few decades, peptide-based therapeutic drugs and peptide-derived technologies have made substantial progress in tumor treatment, targeted delivery, and nanomedicine [3,4,5,6,7]. Currently, over 100 types of peptide drugs have been approved for the market, addressing metabolic diseases, infectious diseases, and various types of tumors [8]. The molecular weight of peptide drugs lies between that of small-molecule compounds (<500 Da) and biologics (>10 kDa), and this unique structural range confers both the tissue permeability of small molecules and the targeted recognition ability of large molecules [9]. Compared to conventional chemotherapeutic agents, peptide drugs exhibit superior pharmacokinetic profiles, enhanced biocompatibility, and reduced immunogenicity. Additionally, their ease of synthesis and structural modifiability enable them to interact with protein interfaces traditionally considered “undruggable” by standard therapeutics, thereby expanding the scope of precision medicine approaches [10,11].

In recent years, malignant neoplasms of the digestive system have emerged as major contributors to the global cancer burden. According to the latest estimates from the Global Cancer Observatory (GCO), approximately 20 million new cancer cases and 9.7 million cancer-related deaths occurred worldwide in 2022. Digestive system malignancies, including colorectal cancer (CRC), gastric cancer (GC), pancreatic ductal adenocarcinoma (PDAC), hepatocellular carcinoma (HCC) and esophageal cancer (EC), account for a large proportion of global cancer incidence and mortality. For example, CRC alone accounted for approximately 1.9 million new cases and over 900,000 deaths worldwide, ranking as the third most diagnosed cancer and the second leading cause of cancer-related mortality globally. These statistics highlight the significant public health burden imposed by digestive system cancers and underscore the urgent need for more effective and targeted therapeutic strategies [12,13]. The current clinical treatments of digestive tract tumors still primarily rely on surgery, radiotherapy, and chemotherapy. However, these traditional methods generally suffer from limited targeting, strong toxicity and side effects, and a high incidence of drug resistance, so more specific and safer alternative solutions are urgently needed [14,15,16]. The occurrence and progression of digestive system malignant tumors are closely related to the abnormal expression of oncogenes and the continuous activation of signaling pathways. Consequently, screening tumor-related genes or identifying peptides that can specifically bind to tumor regulatory factors has become a research hotspot in the field of anticancer drug development [9,17].

Various peptide drugs have demonstrated significant anti-tumor potential across multiple dimensions. The plant-derived peptide lunasin (derived from soybeans) can selectively inhibit the growth of gastrointestinal tumor cells by inducing mitochondrial damage, DNA breakage, and modulating antioxidant/anti-inflammatory pathways. It also exhibits synergistic effects when combined with chemotherapeutic drugs [18]. The peptide Buforin I/II, derived from the gastric tissue of Asian toads, has been shown to upregulate epithelial markers, inhibit epithelial–mesenchymal transition (EMT), and promote CD8^+^ T cell infiltration, thereby suppressing metastasis HCC and PDAC [19,20,21]. Additionally, peptide–drug conjugates (PDCs) facilitate the targeted delivery of cytotoxic agents, such as doxorubicin (DOX) and paclitaxel (PTX), to tumor cells via degradable linkers, resulting in enhanced antitumor efficacy coupled with reduced toxicity [22]. Furthermore, peptide drug delivery systems integrated with nanocarriers, including liposomes and polymeric nanoparticles, substantially improve the stability, plasma half-life, and tissue permeability of peptide drugs, thereby advancing their potential for clinical translation [23].

Collectively, peptide drugs, characterized by their multi-targeting capabilities and structural modifiability, offer promising avenues to overcome the limitations inherent in conventional treatments for malignant tumors of the digestive system. Nevertheless, comprehensive evaluations of the drug development processes and clinical applications of peptides in managing these malignancies remain notably lacking. Existing reviews of peptide therapeutics predominantly focus on discussions of molecular structural modifications or generalized targeting strategies, with limited in-depth exploration of the suitability and translational challenges of different approaches within specific tumor types [22,24,25,26,27,28,29]. To this end, this paper adopts gastrointestinal tumors such as CRC, GC, PDAC, and HCC as the entry point, and reclassifies peptide drugs based on their mechanisms of action. Through a critical review of representative strategies from laboratory mechanisms to clinical bottlenecks, this paper aims to identify key factors influencing development in this field and provide a more targeted reference framework for future research and clinical design.

## 2. Mechanism of Action of Peptide Drugs

The anti-tumor effects of peptide drugs in malignant tumors of the digestive tract are primarily achieved by inducing tumor cell apoptosis, inhibiting proliferation, blocking angiogenesis, reversing multidrug resistance (MDR), and regulating the immune microenvironment [30,31]. BH3 mimetic peptides have been reported to induce programmed cell death by binding to the anti-apoptotic proteins of the Bcl-2 family [32]. The peptide targeting GPC3 in HCC has been shown to achieve targeted delivery by binding with high affinity to tumor cell-specific receptors [33]. In addition, PDC can guide the accurate release of cytotoxic drugs, thus significantly improving the therapeutic efficacy and reducing systemic toxicity [22,34]. Based on their primary mechanisms of action, therapeutic peptides can be classified into three distinct categories: (1) blocking peptides, which disrupt critical oncogenic interactions; (2) delivery peptides, which facilitate the specific transport of therapeutic cargo to tumor sites; and (3) mimetic peptides, which directly modulate cellular signaling pathways by mimicking or blocking endogenous biomolecules [25,35,36].

### 2.1. Blocking Peptides

A blocking peptide functions through structural mimicry or competitive binding. It inhibits the interaction between target proteins and their binding partners by binding to the target proteins with high affinity, thus inhibiting the intermolecular regulatory effects that depend on the interaction [36]. Studies have shown that protein–protein interactions (PPIs) are often dominated by a relatively small number of key amino acid residues at the binding interface. Consequently, peptide segments derived from these interface regions can competitively bind to protein–protein complexes, disrupting the corresponding interactions and inhibiting their biological function [37,38]. Such binding peptides can be systematically derived from the key structural regions of natural binding proteins or obtained through the screening of combinatorial peptide libraries [39]. Furthermore, using structural data and molecular modeling, existing peptides can be rationally optimized, and even a novel binding peptide segment can be designed [40].

Proteins of the Bcl-2 family are anti-apoptotic proteins that regulate cell death by binding to the α-helices of the BH3 (borane) domains in a class of pro-apoptotic proteins, including Bak, Bad, Bid, and Bax. This PPI is frequently impaired in various cancers. Two olefin hydrocarbon staple (HBS) constrained peptides, constructed based on the conserved α-helix in the BH3 domain of Bak, exhibit extremely high stability against proteases, with their stability being about 60 times greater than that of the original peptide [41]. Sulanemadlin (ALRN-6924) is the first cell-permeable α-helical peptidomimetic to enter clinical trials. By simulating the N-terminal domain of the p53 tumor suppressor protein, it binds with high affinity to the endogenous p53 inhibitors MDM2 and MDM4, thereby activating the p53 signaling pathway in cells [42].

### 2.2. Delivery Peptides

Delivery peptides are engineered to specifically identify and bind to molecules that are preferentially overexpressed on tumor cells or within the tumor microenvironment. Their principal function is to serve as targeting moieties, facilitating the conjugation or encapsulation of therapeutic agents (such as drugs or nanoparticles) to enable site-specific accumulation and improved intracellular delivery, thereby reducing off-target effects [25]. These peptides are usually applied as “guiding ligands” in drug delivery systems, including tumor homing peptides, cell-penetrating peptides (CPPs), and PDC [43]. They are endocytosed into the cell by binding to highly expressed, specific receptors on tumor cell membranes or tumor vascular endothelial cells, ensuring precise delivery and deep penetration into tumor tissues and overcoming extracellular matrix barriers, thereby leading to selective accumulation of drugs in tumor tissues and subsequent intracellular release [44].

RGD (Arg-Gly-Asp) peptides target integrins that are overexpressed on the surfaces of tumor cells and tumor vessels, such as αvβ3 and αvβ5 [45]. Similarly, iRGD peptides containing tumor penetration motifs bind to integrins and then interact with neuropilin-1 (NRP-1), thereby promoting the tissue penetration and intracellular delivery of anticancer drugs [46]. Nucleolin is a marker overexpressed on tumor cells and angiogenic vessels, and the F3 peptide can bind to nucleolin to enhance the specific absorption of therapeutic drugs by cancer tissues [47]. The above research mechanisms ensure selective targeting and effective therapeutic outcomes while minimizing the impact on healthy cells.

### 2.3. Mimetic Peptides

Mimetic peptides are designed to mimic the structure of endogenous biomolecules (such as hormones, growth factors, or neuropeptides). They exert therapeutic effects by binding to corresponding receptors, thereby selectively activating or inhibiting key signaling pathways [9]. These peptides are mostly derived from plant or animal proteins and are either extracted from natural sources or chemically synthesized to mimic the structure of natural hormones [35]. In anti-tumor therapy, they can function as growth factors, hormones, neurotransmitters, ion channel modulators, or anti-infective agents, among other functions [48].

Mimetic peptides can mimic endogenous bioligands in cancer therapy, such as growth factors, hormones, or neuropeptides, by binding to corresponding receptors. This interaction selectively activates or inhibits crucial signaling pathways, including PI3K/AKT, MAPK, and JAK/STAT, thereby finely regulating the proliferation, differentiation, and apoptosis of tumor cells [35,49]. In terms of immune regulation, tumor-associated antigen peptides or their analogs can be processed and presented by antigen-presenting cells, thereby inducing or enhancing tumor-specific immune responses through specific interactions with T-cell receptor (TCR) via Major Histocompatibility Complex (MHC) molecules. In addition, some mimetic peptides can simulate the binding domains of antibodies, selectively recognizing tumor-associated molecules, thereby regulating the immune microenvironment or directly mediating immune killing effects. These mechanisms are similar to the modes of action of biological ligands and antigens (such as antibodies and therapeutic proteins), thus providing high specificity and precision in treatment strategies [50].

dTCAPF is a novel hormone peptide that acts through the Toll/interleukin-1 receptor (TIR) signaling pathway, and clinical studies have confirmed its safety and efficacy in the treatment of patients with prostate cancer, HCC, and metastatic cancer. Its anticancer activity may be related to the inhibition of angiogenesis factors, activation of anticancer immune cell function, and induction of endoplasmic reticulum stress [51]. Additionally, five bioactive peptides derived from common legumes (GLTSK, LSGNK, GEGSGA, MPACGSS, and MTEEY) exhibit antiproliferative effects against human colon cancer cell lines (HCT-116, RKO, and KM12L4) and affect the activity of enzymes related to cell proliferation, thereby inducing cell cycle arrest and promoting apoptosis [52].

Peptide drugs used in treating digestive tract malignancies typically act through multiple mechanisms, exhibiting multi-target and multi-pathway synergistic effects. Peptides can form multifunctional systems through structural recombination or coupling, achieving the integration of delivery, recognition, and treatment [53,54]. PDC and peptide-modified nanocarriers can combine specific recognition with cytotoxic effects, significantly improving anti-tumor efficacy and reducing toxicity in animal models. Some peptide drugs can also synergize with immune checkpoint inhibitors, chemotherapy, or radiotherapy, playing a role in combination therapy [55,56].

## 3. Peptide Drugs in the Treatment of Gastrointestinal Malignant Tumors

### 3.1. Colorectal Cancer

According to the latest GCO data, CRC ranks as the third most common cancer globally and the second leading cause of cancer-related deaths [13,57,58]. Genetics, environment, and lifestyle factors are all significant contributors to CRC pathogenesis [59]. CRC exhibits high genetic susceptibility and recurrence tendency, often accompanied by poor prognosis [60]. Peptide-based strategies for CRC have been extensively explored, including tumor-associated antigen vaccines, PDCs, and peptides targeting oncogenic pathways such as KRAS or Wnt signaling.

Blocking peptides are increasingly being investigated in CRC for their ability to competitively disrupt key molecular interactions involved in tumor growth and metastasis. For example, an 11-aa peptide derived from Tiam1 fused with the TAT cell penetrating sequence (TAT-Inh) was developed to block the NSD2-Tiam1 interaction. NSD2-mediated methylation of Tiam1 activates the Rac1-PAK signaling pathway, promoting EMT and metastasis in CRC cells. By competitively disrupting this interaction, TAT-Inh significantly reduces Tiam1 methylation, Rac1-GTP activity, and EMT phenotypes, thereby inhibiting metastatic behavior both in vitro and in vivo [61,62]. Another inhibitory peptide, Lunasin, is a 43-amino acid peptide derived from soybeans containing an RGD motif and a polyaspartic acid tail. It exerts anticancer effects through epigenetic regulation [63,64,65,66]. Lunasin competitively binds to histone acetyltransferases such as p300/CBP-associated factors, reducing histone H3 and H4 acetylation, which inhibits tumor cell proliferation and transformation [35,67,68,69,70,71,72]. These findings highlight the ability of blocking peptides to directly regulate carcinogenic signaling pathways and epigenetic mechanisms in CRC.

In CRC research, delivery peptides primarily function as tumor-homing ligands, facilitating the targeted transport of chemotherapeutic agents or nanocarriers to tumor tissues to enhance intracellular drug accumulation. The laminin-derived pentapeptide YIGSR (Tyr-Ile-Gly-Ser-Arg) exhibits high-affinity binding to the 67-kDa laminin receptor (67LR), which is highly expressed in metastatic CRC cell lines such as HCT-116 [73]. By exploiting this targeting mechanism, YIGSR has been incorporated into drug delivery systems. For instance, the peptide was conjugated to gemcitabine (GEM) via a degradable linker and encapsulated within carboxymethyl chitin/polyamine tree nanoparticles. Through YIGSR-mediated receptor recognition, this system significantly enhances GEM uptake in CRC cells, improves intracellular drug accumulation, and overcomes limitations posed by non-specific distribution and insufficient blood concentrations. Both in vitro and in vivo experiments demonstrated enhanced cytotoxicity, tumor growth inhibition, and favorable safety profiles [74,75].

**Figure 1:**
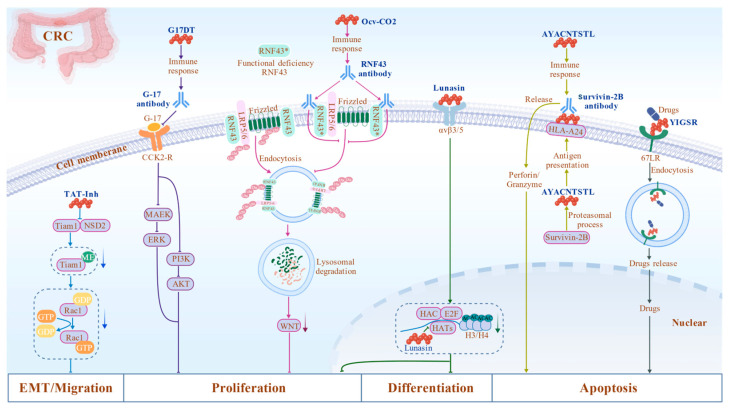
Representative mechanisms of peptide therapeutics in CRC. Blocking peptides (e.g., TAT-Inh, Lunasin) modulate epigenetic regulation or inhibit tumor progression by disrupting oncogenic signaling pathways such as NSD2-Tiam1 and histone acetyltransferases. Delivery peptides (e.g., YIGSR) enhance receptor-mediated endocytosis and facilitate intracellular drug accumulation. Mimetic peptides (e.g., Ocv-C02, G17DT, and Survivin-2B epitope peptide) activate tumor-specific immune responses, ultimately suppressing proliferation and promoting apoptosis in CRC cells. Peptide and antibody names are shown in blue, and colored lines indicate distinct peptide pathways (gray lines represent shared pathways). Created with BioGDP.com [89].

Mimetic peptides are primarily designed to regulate CRC cell signaling or activate tumor-specific immune responses by mimicking endogenous biomolecules or tumor antigens. The anti-gastrin-17 (anti-G-17) immunogen G17DT is a synthetic G-17 derivative peptide vaccine that induces neutralizing antibodies against gastrin. Gastrin and its precursors promote colorectal tumor growth by activating the cholecystokinin-2 receptor (CCK2-R). Antibodies generated by G17DT block progesterone binding to CCK2-R, thereby inhibiting tumor cell proliferation and promoting apoptosis [76,77,78,79]. Mimetic peptides are also employed as tumor vaccines to stimulate antitumor immunity. Peptide vaccines targeting tumor-associated antigens, such as RNF43/TOMM34 (OCV-C02) or Survivin-2B epitopes, can be presented by antigen-presenting cells and recognized by cytotoxic T lymphocytes (CTLs). Clinical studies indicate that RNF43 and TOMM34-derived peptides, when combined with adjuvant chemotherapy, induce tumor-specific immune responses and improve recurrence-free survival in colorectal cancer patients [80,81,82,83]. The Survivin-2B epitope peptide vaccine (AYACNTSTL) stimulates HLA-A24-restricted CTL responses that recognize survivin-expressing tumor cells and induce apoptosis via perforin- and granzyme-mediated mechanisms [84,85,86,87,88]. These diverse mechanisms of action together form a potential therapeutic network for peptide drugs in the treatment of CRC (Figure 1).

Peptide-based therapeutic strategies for CRC encompass diverse research directions, each exhibiting distinct advantages and limitations in terms of mechanism characteristics and clinical translation potential. For instance, TAT-derived inhibitory peptides targeting the Tiam1-NSD2 axis enable intracellular delivery by crossing cell membranes, overcoming a key limitation of traditional peptides that struggle to penetrate cells [61]. These intracellular inhibitory peptides exert effects by disrupting interactions between oncogenic proteins, offering the advantage of targeting protein complexes or signaling nodes that are difficult for conventional small-molecule drugs to effectively modulate. This strategy holds potential for blocking signaling networks associated with tumor invasion and metastasis. Naturally occurring bioactive peptides represent another research avenue. For instance, Lunasin, derived from soybeans, has garnered attention due to its natural origin and widespread dietary presence [90,91]. These characteristics suggest potential safety advantages, positioning it as a candidate for chemoprevention or adjuvant therapy. However, it should be noted that the antitumor activity observed in experimental models typically requires high concentrations, which are often difficult to achieve through conventional dietary intake or systemic administration. This limitation partially restricts their clinical application prospects. In the field of immunotherapy, the peptide vaccine OCV-C02, targeting RNF43/TOMM34, has entered Phase I clinical trials. Results indicate this vaccine exhibits acceptable safety and induces detectable cytotoxic T cell responses in some patients, particularly those carrying specific HLA genotypes such as HLA-A*24:02 [80,92]. Concurrently, peptide-directed drug delivery systems are gaining attention. For instance, the PDC YIGSR-GEM enhances chemotherapy drug accumulation in tumor tissues via receptor-binding peptides [74,93]. Compared to conventional chemotherapy, these strategies offer promise for improving the therapeutic index by enhancing tumor targeting and reducing exposure to normal tissues. Preclinical studies have demonstrated that these constructs can enhance intracellular drug delivery and increase cytotoxic effects on tumor cells.

### 3.2. Gastric Cancer

According to the latest data from the GCO, GC remains a major global health challenge [13]. Risk factors for this disease include *Helicobacter pylori* (*H. pylori*) infection, age, high salt intake, and low fruit and vegetable consumption [94]. Despite its limitations, surgical or endoscopic resection remains an indispensable cornerstone of treatment. Currently, partial progress has been made with molecular targeted therapies against specific targets, but overall efficacy for advanced GC remains limited. Furthermore, challenges related to drug resistance and systemic toxicity persistently hinder treatment outcomes [95,96]. GC peptide research primarily focuses on tumor antigen-derived peptide vaccines and peptides targeting angiogenesis.

Peptides targeting oncogenic signaling pathways or resistance mechanisms demonstrate promising potential in GC therapy. Given that *H. pylori* infection is a primary etiology, peptides targeting this infection or tumor-associated pathways are also under investigation [97,98,,99]. For example, the bioactive peptide H-P-6 (PQPKVLDS), derived from microbial hydrolysates, inhibits *H. pylori*-associated gastric cancer by suppressing epidermal growth factor receptor (EGFR) activation and downregulating the PI3K/Akt signaling pathway, while also preventing β-catenin nuclear translocation [100]. Other blocking peptides may overcome MDR in GC, such as the GRP78-targeting peptide GMBP1 (ETAPLSTMLSPY) identified via phage display screening. This peptide enters multidrug-resistant GC cells via clathrin-mediated endocytosis and inhibits GRP78 expression by targeting the PI3K/AKT-EIF4E pathway, thereby downregulating MDR1 expression. This reverses chemotherapy resistance and restores sensitivity to drugs like cisplatin and DOX in resistant GC cell lines [101,102].

Delivery peptides are extensively used in GC to guide drugs, imaging probes, or therapeutic proteins to tumor vasculature or endothelial cells. The aforementioned GRP78-targeting peptide GMBP1 can deliver chemotherapeutic agents. Additionally, the GX1 peptide participates in GC-associated angiogenesis and serves as a targeting ligand for tumor vascular imaging and therapy [103,104,105]. For example, GX1-PTX-NLCs is a nanostructured lipid carrier system loaded with PTX and conjugated with GX1 (sequence CGNSNPKSC), which disrupts tumor vascular endothelium and inhibits neovascularization, thereby indirectly suppressing tumor cell proliferation. Compared to free PTX, this system exhibits stronger inhibitory effects on co-cultured human umbilical vein endothelial cells with lower cytotoxicity toward normal cells, while providing sustained drug release and enhanced drug accumulation within tumor vasculature [106,107]. Similarly, the TCP-1 peptide has been utilized to deliver tumor necrosis factor-α (TNF-α) specifically to tumor vessels. The TCP-1/TNF-α fusion protein promotes vascular normalization and enhances intratumoral chemotherapy drug perfusion, thereby promoting apoptosis and inhibiting GC xenograft growth when combined with 5-fluorouracil (5-FU) [108]. Another vascular-targeting peptide, GEBP11, selectively binds to endothelial cells and tumor vessels and has been widely used for targeted imaging and drug delivery. GEBP11 has been conjugated with magnetic nanoparticles or radionuclides to enable multimodal imaging, including MRI and fluorescence imaging, for in vivo detection of GC vasculature [109,110]. Fluorescent probes constructed by linking GEBP11 with dyes such as Cy5.5 (Cyanine 5.5) allow precise visualization of tumor margins and micrometastases in animal models, aiding surgical resection [111]. Furthermore, conjugation with radionuclides like iodine-131 enables targeted radiotherapy to tumor vasculature, significantly suppressing tumor growth and prolonging survival in animal studies [112]. Structural modifications, including PEGylation or trimerization, further enhance tumor accumulation and retention rates, supporting its potential as a targeted delivery platform for anti-angiogenic drugs [113].

**Figure 2:**
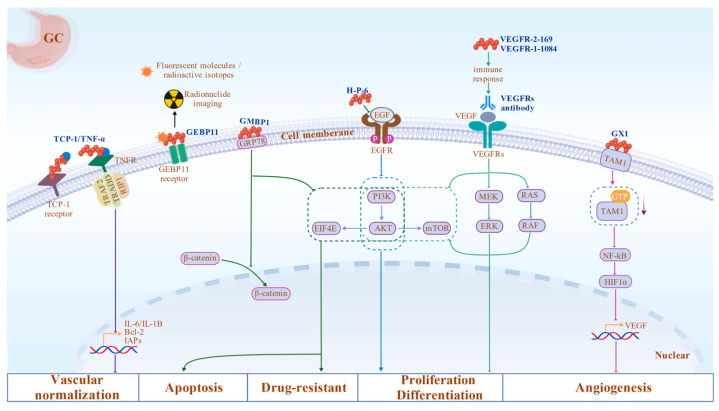
Representative mechanisms of peptide therapeutics in GC. Blocking peptides (e.g., H-P-6, GMBP1, GX1) inhibit tumor growth by targeting pathways associated with *H. pylori*, GRP78-mediated PI3K/AKT signaling, and angiogenesis. Delivery and mimetic peptides (e.g., GEBP11, VEGFR1-1084/VEGFR2-169 vaccine, and TCP-1/TNF-α) target tumor vasculature or endothelial cells to enable targeted drug delivery, inhibit angiogenesis, and suppress GC progression. Peptide or antibody names are shown in blue, and colored lines indicate distinct peptide pathways (gray lines represent shared pathways). Created with BioGDP.com.

Immunotherapeutic strategies targeting tumor vasculature are currently under investigation. The VEGF/VEGFR axis is indispensable during angiogenesis and is considered a key driver of tumor angiogenesis [114]. Activation of VEGFRs can be mediated by ligand binding. Subsequently, ligand-induced conformational changes in the VEGFR intracellular domain promote receptor dimerization, leading to autophosphorylation of specific tyrosine residues and activating multiple downstream enzyme pathways, including p38/MAPK, RAS/RAF/MEK/ERK, and PI3K/AKT/mTOR [115]. In clinical studies, vaccines composed of cancer–testis antigens (FOXM1, URLC10, KIF20, DEPDC1) and VEGFR-derived peptides successfully induce antigen-specific CTL responses in advanced GC patients, thereby inhibiting tumor angiogenesis and suppressing tumor growth [116]. Notably, the clinical utilization of anti-VEGF monoclonal antibodies is substantially constrained by the inhibition of physiological angiogenesis, a prevalent adverse effect associated with anti-angiogenic treatment [117]. These strategies collectively highlight the precise targeting capability of peptide drugs in the treatment of GC (Figure 2).

In GC, peptide vaccines represent one of the major translational approaches, with the primary objective of eliciting tumor-specific immune responses. However, the overall clinical benefit of these vaccines remains difficult to determine. Early clinical studies have generally involved small patient cohorts and frequently lack randomized controlled designs, which limits the ability to accurately assess therapeutic efficacy. For example, the URLC10/VEGFR1 peptide vaccine has demonstrated acceptable safety and immunogenicity in early clinical trials, yet the induction of antigen-specific immune responses has not consistently translated into significant improvements in patient survival. This discrepancy highlights a common challenge in peptide-based immunotherapy, where measurable immune activation does not necessarily correlate with durable clinical benefit [118,119]. In addition, the requirement for specific HLA genotypes may further restrict the proportion of patients eligible for such vaccine-based therapies. Targeting peptides have also shown promise in drug delivery applications in GC. Peptides such as GX1 and GMBP1 possess defined targeting mechanisms and strong tumor-binding capacity, and they are therefore frequently incorporated as targeting ligands in nanoparticle-based drug delivery systems to enhance drug accumulation at tumor sites [104,120,121].

### 3.3. Pancreatic Ductal Adenocarcinoma

PDAC represents a highly lethal malignancy with a rapidly increasing global burden. According to data from the GCO, pancreatic cancer accounted for more than 500,000 new cases worldwide in 2022 [13]. The current standard treatment primarily relies on surgical resection combined with adjuvant chemotherapy. Nevertheless, due to its insidious onset, most patients are diagnosed at an advanced stage, resulting in suboptimal therapeutic outcomes [122]. Therefore, peptide-based PDAC strategies primarily aim to inhibit KRAS signaling, enhance tumor permeability (using iRGD peptides), or improve targeted drug delivery.

In PDAC research, blocking peptides primarily target oncogenic signaling pathways such as KRAS-MAPK or Wnt/β-catenin to inhibit tumor growth and metastasis. Peptides derived from the WW domain of IQGAP1 competitively bind ERK1/2 and disrupt the IQGAP1-ERK complex, thereby inhibiting ERK activation and inducing apoptosis in PDAC cells [123,124,125,126,127,128]. peptides CP-FaP2 and CP-FaP3 disrupt FAM83A-β-catenin interactions, preventing β-catenin stabilization and blocking sustained Wnt signaling activation [129]. Peptides blocking the STYK1-β-catenin or STYK1-GSK3β interfaces also inhibit PDAC progression by restoring β-catenin degradation and suppressing tumor cell proliferation [129,130,131]. Another bifunctional peptide, PEP-010, is a dual-acting pro-apoptotic peptide capable of penetrating tumor cells and disrupting key intracellular regulatory complexes. Mechanistically, PEP-010 specifically disrupts the interaction between caspase-9 and protein phosphatase 2A (PP2A), thereby releasing caspase-9 from the inhibitory control of PP2A and activating downstream apoptotic signaling. This process subsequently leads to the activation of caspase-3, PARP cleavage, and widespread apoptosis in tumor cells [132]. Preclinical studies demonstrate that PEP-010 alone significantly inhibits tumor growth in patient-derived xenograft models. Its antitumor activity is further enhanced when combined with chemotherapeutic agents such as PTX or GEM, producing synergistic therapeutic effects [133,134]. These findings suggest that peptides targeting intracellular regulatory complexes represent a promising approach to restoring apoptotic signaling in PDAC cells, which are inherently resistant to conventional therapies.

Peptide delivery represents another critical research direction in PDAC therapy, aimed at overcoming the dense stromal barrier and poor drug penetration inherent in this malignancy. The tumor-penetrating peptide iRGD is among the most extensively studied examples. It operates through a two-step targeting mechanism. The peptide first binds to integrin αvβ3/β5 expressed on tumor vessels, followed by proteolytic cleavage that reveals the CendR motif, which activates NRP-1-mediated tissue penetration. This process transiently opens transport pathways within tumor vessels, significantly enhancing the penetration and distribution of co-administered drugs within the dense PDAC matrix [116,135,136,137,138,139,140,141]. Preclinical studies demonstrate that combining iRGD with chemotherapy regimens such as GEM and nab-PTX markedly improves drug delivery efficiency and therapeutic outcomes [142,143]. Another delivery peptide, LyP-1, which shares a penetration mechanism similar to IRGD, binds to the p32 receptor expressed on tumor cells and tumor-associated macrophages. This promotes receptor-mediated endocytosis and enhances tumor tissue penetration. LyP-1 has been incorporated into functionalized imaging probes and nanoparticle drug carriers, significantly improving tumor accumulation and enabling more precise imaging and drug delivery in PDAC models [144,145,146,147]. PTP peptides specifically recognize the PDAC-associated membrane protein plectin-1 and have been utilized to construct redox-responsive nanocarrier systems capable of co-delivering PTX and therapeutic siRNA. Within the glutathione-rich tumor microenvironment, the nanocarrier undergoes dissociation, triggering payload release and inducing apoptosis by inhibiting anti-apoptotic proteins such as Bcl-2 and Survivin and activating caspase-3 signaling [148,149,150].

**Figure 3:**
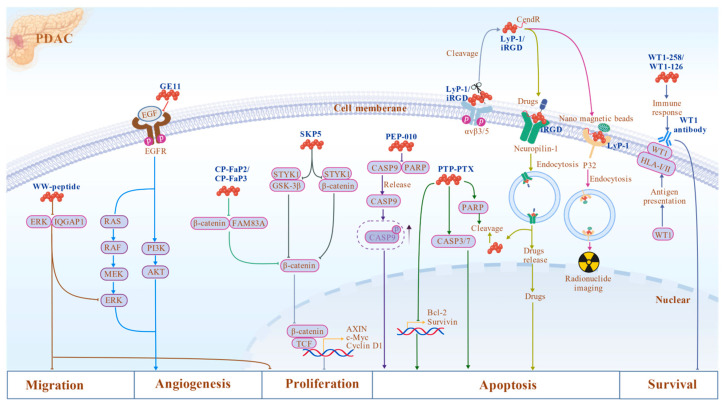
Representative mechanisms of peptide therapeutics in PDAC. Blocking peptides (e.g., WW peptide, CP-FaP2/CP-FaP3, SKP5, and PEP-010) inhibit tumor progression by disrupting oncogenic signaling pathways such as ERK, Wnt, and caspase-9/PP2A interactions. Delivery peptides (e.g., iRGD and LyP-1) enhance tumor targeting and tissue penetration through integrin/NRP-1-mediated transport or receptor-mediated endocytosis. Mimetic peptides (e.g., WT1 peptide vaccines and GE11) regulate tumor signaling or activate tumor-specific immune responses, thereby suppressing tumor growth and promoting apoptosis. Peptide or antibody names are shown in blue, and colored lines indicate distinct peptide pathways (gray lines represent shared pathways). Created with BioGDP.com.

Research on mimetic peptides has also been extensive in PDAC therapy. GE11 peptide serves as a key example, selectively binding to the EGFR without activating its mitotic signaling. Given the frequent overexpression of EGFR in pancreatic tumors, GE11 is widely employed as a targeting ligand to modify nanocarriers or micellar systems for selective drug delivery to EGFR-positive PDAC cells. GE11-modified carriers significantly enhance the stability, targeting precision, and intracellular uptake of chemotherapeutic agents such as GEM, thereby improving antitumor efficacy while reducing off-target toxicity [151,152,153,154]. Beyond directly targeting signaling proteins, strategies focusing on the tumor microenvironment are also being explored. For instance, the vaccine G17DT induces the production of high-titer neutralizing antibodies against G-17, blocking G-17-mediated activation of the MAPK/ERK and PI3K/AKT pathways via the CCK2-R, thereby inhibiting PDAC cell proliferation and inducing apoptosis [155]. Preclinical studies have shown significant survival extension, and its specificity has led to orphan drug designation in the United States, the European Union, and Australia [156,157]. WT1 peptide vaccines target the Wilms tumor 1 (WT1) antigen, which is highly expressed in approximately 70–80% of PDAC tissues and correlates with poor prognosis [158]. These peptide vaccines deliver WT1 epitopes to antigen-presenting cells, enabling their presentation via HLA class I and II molecules, thereby inducing WT1-specific CD8^+^ cytotoxic T lymphocyte and CD4^+^ helper T cell responses. The resulting immune activation generates tumor-specific immune memory capable of eliminating WT1-expressing tumor cells [159,160,161,162]. Clinical studies indicate that combining WT1 peptide vaccines with standard chemotherapy regimens such as GEM, S-1, or nab-PTX prolongs progression-free and overall survival in certain patient cohorts, particularly those exhibiting robust WT1-specific immune responses [163,164,165,166,167]. Peptide-based immunotherapy strategies may complement conventional treatments, promoting more effective PDAC management. Collectively, these mechanisms illustrate the synergistic effects of peptide drugs against PDAC (Figure 3).

PDAC presents the greatest demand yet faces the most significant challenges in the field of peptide therapy. Drug delivery in PDAC remains particularly challenging due to the dense stromal architecture and highly heterogeneous tumor microenvironment. Tumor-penetrating peptides such as iRGD have attracted significant interest because of their ability to facilitate drug penetration into otherwise poorly accessible tumor regions. Experimental studies have shown that iRGD can improve intratumoral drug distribution and enhance the antitumor efficacy of co-administered chemotherapeutic agents [168]. However, the effectiveness of this strategy appears to depend partly on the expression of NRP-1, which exhibits substantial variability among PDAC tumors and may therefore limit the general applicability of iRGD-mediated therapies [169,170]. In addition to iRGD, other receptor-targeting peptides, including WW peptides and GE11, have demonstrated considerable activity in experimental models characterized by high receptor expression [171,172]. The WT1 peptide vaccine has shown favorable safety and immunogenicity profiles in several phase I/II clinical studies [173,174].

### 3.4. Hepatocellular Carcinoma

Globally, HCC is a major contributor to the global cancer burden. Data from the GCO indicate that over 900,000 new liver cancer cases and more than 830,000 deaths were recorded in 2022, highlighting the aggressive nature and poor prognosis of this malignancy [13]. The development of HCC is closely associated with chronic hepatitis B or C virus infection, alcoholic cirrhosis, and non-alcoholic fatty liver disease. Most patients are diagnosed at an advanced stage, and the disease is prone to recurrence or metastasis [175,176]. Major therapeutic challenges include drug resistance, toxicity, and tumor heterogeneity [177,178]. In HCC, peptide development primarily involves receptor-targeting peptides and CPP-based delivery systems.

Blocking peptides represent a significant direction in HCC research. The peptide FFW targets the interaction between SALL4 and the nucleolar remodeling and deacetylase (NuRD) complex. SALL4 is a tumor-associated fetal transcription factor that is reactivated in numerous HCC cases and is closely associated with tumor progression and poor prognosis. By disrupting the SALL4-NuRD interaction, the FFW peptide alleviates transcriptional suppression of tumor suppressor genes and converts SALL4 from a dual-function transcription factor to a single-function activator. This shift ultimately induces apoptosis and inhibits migration in SALL4-positive hepatocytes, while demonstrating significant antitumor efficacy in mouse tumor models [179]. The C7 peptide, which functions as a competitive antagonist of hepatocyte growth factor (HGF), prevents HGF from binding to the c-Met receptor. By blocking the HGF/c-Met signaling axis and downstream pathways such as PI3K/Akt and Erk1/2 activation, C7 significantly inhibits HCC cell invasion, metastasis, and angiogenesis activity, thereby reducing tumor progression in experimental models [180].

Mimetic peptides represent another therapeutic strategy explored in HCC. As an example, HM-3, an RGD-containing peptide, is designed to inhibit the activation of integrin αvβ3 signaling. Integrins play a pivotal role in mediating endothelial cell adhesion, migration, and angiogenesis within the tumor microenvironment. By binding to integrin αvβ3 on endothelial cells, HM-3 inhibits integrin-mediated signaling pathways, disrupting the angiogenesis process essential for tumor vascular formation. Consequently, HM-3 suppresses endothelial cell migration and angiogenesis while reducing metastatic potential in tumor models [181]. Preclinical studies indicate this peptide exhibits significant antitumor activity by inhibiting the ERK and AKT signaling pathways downstream of integrin activation, thereby impairing tumor growth and angiogenesis [181,182]. Despite promising anti-angiogenic effects demonstrated in experimental studies, the clinical translation of HM-3 remains limited due to pharmacokinetic constraints observed in early research, such as a short half-life and a complex dose-response relationship.

Peptide-based therapeutic strategies targeting HCC remain largely in the early exploratory stage. HM-3 and its modified derivatives, including PEG-conjugated and albumin-fused variants, have demonstrated anti-angiogenic activity in preclinical studies. However, their therapeutic advantages relative to established first-line agents such as sorafenib or lenvatinib have not yet been clearly demonstrated [183]. Peptides C7 and FFW exhibit potential in targeting specific oncogenic signaling pathways, although the possibility of resistance mediated by compensatory pathway activation should be considered [180].

### 3.5. Esophageal Cancer

According to the GCO database maintained by the International Agency for Research on Cancer, EC ranks among the top ten cancers globally and is the sixth leading cause of cancer-related mortality. Esophageal squamous cell carcinoma (ESCC) is the predominant histological subtype worldwide, particularly in East Asia and certain regions of Africa [13]. In the field of EC, peptide research is still in its early stages, primarily focusing on tumor-targeting peptides for drug delivery [184], molecular imaging [185], or nanoparticle-based therapeutic systems [186], indicating it remains in an early developmental stage.

Peptide-based blockade strategies are being developed to target numerous oncogenic transcription complexes in ESCC that resist inhibition by conventional small-molecule drugs. The transcription factor SOX2 plays a pivotal role in maintaining cancer stemness and driving ESCC tumor progression. The P58 peptide was engineered to disrupt the interaction between SOX2 and the chromatin-associated protein CDP. By competitively blocking CDP-SOX2 complex formation, P58 inhibits SOX2-mediated transcriptional activity that drives tumor cell proliferation and invasion. This interference results in tumor growth suppression, reduced metastatic potential, and induced apoptosis in experimental models. Importantly, P58 utilizes a cell-penetrating peptide motif for intracellular delivery and effectively inhibits this “undruggable” PPI, offering a promising approach for directly targeting transcription factor-dependent oncogenic signaling in ESCC [187].

**Figure 4:**
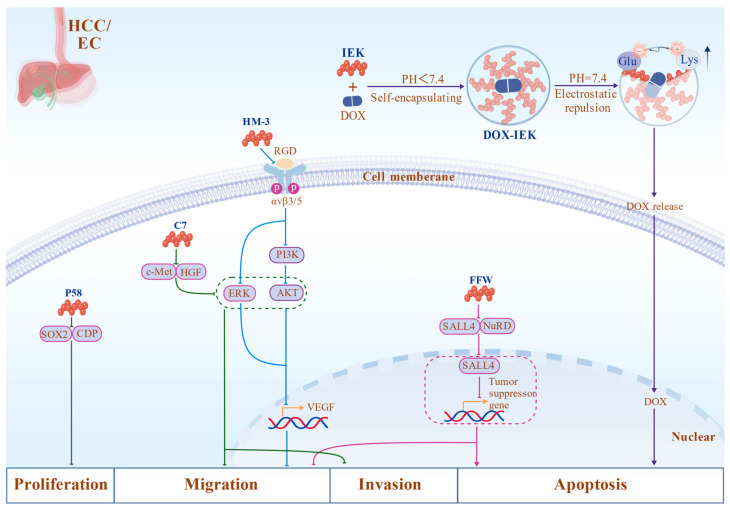
Representative mechanisms of peptide therapeutics in HCC/EC. Blocking peptides (e.g., P58, C7, and FFW) inhibit tumor progression by disrupting oncogenic interactions or signaling pathways such as CDP-SOX2, HGF/c-Met-mediated PI3K/AKT and ERK pathways, and SALL4-associated transcriptional repression. Mimetic peptides (e.g., HM-3) suppress angiogenesis by targeting integrin-mediated endothelial cell migration. Delivery peptides (e.g., IEK) form pH-responsive self-assembled systems that enable controlled drug release within the acidic tumor microenvironment. Peptide or antibody names are shown in blue, and colored lines indicate distinct peptide pathways (gray lines represent shared pathways). Created with BioGDP.com.

The DOX-IEK peptide system, a pH-responsive self-assembling hydrogel, enables controlled delivery of DOX. The IEK peptide sequence self-assembles into nanostructured hydrogels under physiological conditions and responds to the acidic microenvironment typical of tumor tissues. Upon exposure to the lower pH conditions within tumors, the hydrogel structure gradually disintegrates, facilitating localized DOX release and enhancing drug accumulation at the tumor site. This pH-sensitive delivery mechanism not only enhances the antitumor efficacy of chemotherapy but also reduces systemic toxicity and adverse reactions associated with conventional drug administration [188]. Experimental studies demonstrate that such peptide-based delivery systems exhibit promising tumor suppression effects both in vitro and in vivo, highlighting their potential as an innovative platform for targeted therapy in ESCC [189]. These peptide-based innovative approaches offer new strategies for the personalized treatment of EC (Figure 4).

The P58 peptide has been shown to disrupt the interaction between SOX2 and CDP proteins and has demonstrated antitumor activity in experimental studies, suggesting a potential strategy for targeting transcriptional regulatory networks in cancer [187]. In addition, peptide-based delivery materials, such as the DOX-IEK hydrogel system, represent a promising approach for optimizing chemotherapeutic administration. These systems enable pH-responsive and controlled drug release and may reduce systemic toxicity while maintaining antitumor efficacy compared to conventional formulations [189,190].

## 4. Optimization Strategies for Peptide Drugs

Peptide drugs demonstrate promising targeting capabilities and biocompatibility for treating gastrointestinal malignancies. However, their clinical translation is still limited by shortcomings such as poor in vivo stability, susceptibility to proteolytic degradation, short plasma half-life, and low oral bioavailability [191]. In order to address these limitations, research has focused on two primary optimization strategies. First, improvements in delivery routes have been investigated to enhance targeting efficiency and therapeutic outcomes [191,192,193]. Second, chemical modifications to the peptide structure have been explored to increase enzymatic stability and prolong circulation time, thereby optimizing pharmacokinetic properties [194].

### 4.1. Strategies to Improve Delivery Efficiency

Nanodrug delivery systems utilize nanomaterials to load peptide drugs, aiming to enhance therapeutic efficacy by improving delivery efficiency [195]. Among them, self-assembling peptides constitute an important component of nanodrug delivery systems, capable of spontaneously forming ordered nanostructures with high physicochemical stability through intermolecular interactions [196]. For instance, conjugating the RGD peptide to PTX via a succinate linker leads to the self-assembly of nanofibers, which exhibit superior antitumor activity compared to free PTX in GC models [197].

Liposomes are a well-established delivery platform. Their lipid bilayers can self-assemble into hollow vesicles in aqueous environments via hydrophobic interactions, enabling simultaneous encapsulation of hydrophilic drugs in the aqueous core and loading of lipophilic compounds within the membrane [198,199]. Based on this principle, a dendrimeric peptide-based lipopeptide-liposome hybrid platform has been designed, which has demonstrated excellent antitumor efficacy in mitochondria-targeted delivery [200,201].

Extracellular vesicles (EVs) are nanoscale, membrane-bound vesicles secreted endogenously, which naturally transport a diverse array of bioactive molecules, including proteins, peptides, mRNAs, and non-coding RNAs [202,203,204]. EVs are primarily categorized based on their size into exosomes (<150 nm), microvesicles (100–1000 nm), and apoptotic bodies (>1000 nm) [205]. Under pathological conditions, the secretion of EVs by cells is markedly elevated, often accompanied by changes in their size distribution [206,207]. Due to their favorable biocompatibility and intrinsic targeting capabilities, EVs have gained considerable attention as potential delivery vehicles for peptide-based therapeutics. For instance, EVs derived from erythrocytes have been utilized for targeted leukemia therapy [208]. Furthermore, conjugating EVs with peptides and nanobodies has been demonstrated to enhance the specificity and efficiency of targeted delivery.

In addition to optimizing delivery systems, structural modification of peptide molecules is a key strategy for enhancing their stability. Common approaches include cyclization, D-amino acid substitution, PEGylation, and fatty acid conjugation [209].

### 4.2. Strategies to Enhance Stability

Cyclization design enhances the structural stability and target-binding capacity of peptides by reducing conformational freedom through covalent ring closure, such as via disulfide bonds or backbone cyclization. For instance, a comparative study between the linear RGD peptide (C18-ADA5-RGD) and its cyclized analog cRGDfK (C18-ADA5-cRGDfK) evaluated their drug-loading performance in tumors overexpressing αvβ3 integrin. The results showed that the cyclized cRGDfK carrier had a lower critical micelle concentration (CMC: 8 μM vs. 25 μM for the linear form) and exhibited superior cellular binding and uptake efficiency [210]. In an HCC model, the cyclic cRGDfK peptide targeting αvβ3 integrin demonstrated enhanced suppression of EMT and metastasis, further supporting the role of cyclization in improving peptide functional activity [211]. Furthermore, cyclization approaches have demonstrated significant benefits in various target settings. For instance, the linear peptide-peptide hybrid molecule linear 13 exhibited low affinity for the β-catenin/TCF complex due to its high structural flexibility. To overcome this conformational flexibility limitation, researchers employed a macrocyclic strategy to lock its active conformation. The resulting cyclic compound 13 demonstrated approximately 31-fold enhanced activity, significantly inhibiting the proliferation of prostate cancer cells [212].

The incorporation of D-amino acids or non-natural amino acids (e.g., N-methylated amino acids) is an effective strategy to enhance enzymatic stability and prolong the half-life of peptides. Studies have shown that introducing 1-3 D-amino acids at the N- or C-terminus of a MUC2-related epitope peptide resulted in derivatives that remained almost undegraded over 96 h [213]. By employing a D-amino acid substitution approach, which preserves the activity of essential L-amino acids while replacing the others with D-isomers, the resulting CSBP peptide remained stable in serum for over 96 h without degradation [214]. Moreover, N-methylation can markedly improve peptide stability; a single β-alanine amino acid, with a methylated nitrogen, was used as a linker in a statine-based gastrin-releasing peptide receptor (GRPR)-antagonist radiopharmaceutical designed to target receptors on the surface of several human tumors. The introduction of the *N*-methylated β-alanine did not disrupt the binding affinity and presented a similar in vivo stability in mice compared to the unmodified compound [215]. β-Amino acids significantly improve metabolic stability by altering the geometric structure of the peptide backbone and disrupting the hydrogen bond network required for protease cleavage, thereby making peptides less susceptible to recognition and hydrolysis by proteases in vivo [216]. Bombesin is a neuropeptide with potential for targeting breast and prostate tumors. It is rapidly metabolized in vivo and exhibits significantly prolonged plasma half-life by introducing β-amino acids into the bombesin peptide chain, while maintaining high affinity for the GRPR. Its excellent in vivo stability and tumor uptake capacity have been validated in mouse models [217].

PEGylation is a widely used chemical strategy for engineering peptide-based therapeutics. PEG is characterized by its biodegradability, low toxicity, and low immunogenicity. Conjugating PEG to peptides can significantly enhance drug stability and prolong plasma half-life through mechanisms such as increasing molecular size, shielding proteolytic sites, and reducing renal clearance. HVGGSSV is a peptide targeting Tax-interacting protein 1 (TIP1), which is highly expressed in cancer cells. Docking analysis showed a binding energy of −6.0 kcal/mol and a K_D_ of 3.3 × 10^−6^ M. To improve circulation time, the peptide was conjugated with 40 kDa PEG and labeled with ^111^In for SPECT/CT imaging. The PEGylated peptide exhibited a prolonged plasma half-life and specifically accumulated in tumor models in the neck, esophagus, pancreas, lungs, and brain within 48–72 h, demonstrating good in vivo stability and tumor targeting ability [218].

Fatty acid acylation involves covalently attaching long-chain fatty acids to specific sites on peptide drugs, enhancing their pharmaceutical properties and extending their half-life. This modification enhances peptide stability by conformational stabilization, thereby prolonging systemic circulation. Moreover, given the structural similarity between fatty acids and membrane phospholipids, acylated peptides often exhibit increased lipophilicity, thereby improving intestinal absorption and mucosal permeability. Additionally, the incorporated fatty acid moiety can bind to human serum albumin. The resulting complex, due to its increased molecular size, is less susceptible to rapid clearance, further extending the peptide’s residence time in the bloodstream [219]. This strategy has been successfully employed in clinically approved drugs. For instance, the antidiabetic peptide drugs liraglutide and semaglutide both incorporate fatty acid chains, which enhance hydrophobicity, mask the dipeptidyl peptidase-4 cleavage site, reduce renal excretion, and significantly prolong their therapeutic half-lives [220,221].

## 5. Discussion

This review systematically summarizes recent advances in peptide-based therapeutics for major gastrointestinal malignancies, focusing on three functional peptide categories, including blocking peptides, delivery peptides, and mimetic peptides. Collectively, these strategies demonstrate the diverse roles of peptides in cancer therapy (Table 1), such as inhibiting oncogenic signaling pathways, enabling targeted drug delivery, and activating immune response. Notably, peptide delivery systems and peptide-based targeting strategies have contributed the most substantial advances in translational research, while peptide vaccines and partial-blocking peptides remain promising but exploratory.

In summary, the clinical translation of peptide therapeutics continues to face numerous persistent challenges that collectively limit their widespread application. A primary constraint is the inherent instability and unfavorable pharmacokinetic properties of peptides. Peptides are highly susceptible to enzymatic degradation, often exhibiting short plasma half-lives and low bioavailability, which significantly impede their effective accumulation at tumor sites [9,226,227]. While chemical modification strategies such as cyclization, N-terminal or C-terminal modification, or D-amino acid substitution can enhance stability, these modifications may simultaneously alter peptide conformation and reduce targeting affinity, or introduce unintended immunogenicity [228,229]. Solid tumors typically exhibit dense extracellular matrices, high interstitial pressure, and heterogeneous vasculature, all of which constrain the uniform distribution of therapeutic drugs, resulting in “therapeutic blind spots” in the core area of the tumor [230]. Even CPPs, such as the widely used TAT-based system for intracellular delivery, may accumulate non-specifically in normal tissues due to their limited tumor selectivity, raising concerns about off-target toxicity [188,231,232,233,234].

In addition, a clear translational gap remains between experimental research and clinical application. Although several peptide-based strategies have demonstrated promising safety profiles and antitumor activity in early studies, strong clinical evidence supporting their therapeutic benefit is still limited. For instance, the RNF43/TOMM34 peptide vaccine OCV-C02 has shown favorable safety and the ability to induce antitumor immune responses in early clinical trials, but evidence from randomized controlled studies remains insufficient [80,81,82,83]. Regulatory and developmental requirements also influence the clinical advancement of peptide therapeutics. Before clinical approval, comprehensive evaluations of pharmacokinetics, biodistribution, metabolic stability, and long-term toxicity are required, along with ensuring the reproducibility of peptide synthesis and the manufacturing consistency of delivery systems. For platforms incorporating CPPs or self-assembling delivery materials, regulatory agencies often apply stricter quality control standards, including assessments of biodegradability, potential immunotoxicity, and interactions with existing therapies such as sorafenib. Currently, most peptide-based therapies remain in early clinical development, and standardized evaluation frameworks are still evolving, which may affect the pace of their clinical translation [235,236,237,238].

Addressing these challenges requires integrated strategies combining peptide engineering, delivery technologies, and precision oncology. Firstly, rational peptide design leveraging computational modeling, structural biology, and artificial intelligence can enhance structural stability and selectivity, thereby simultaneously improving pharmacokinetic properties and target affinity. Secondly, developing tumor microenvironment-responsive delivery systems represents a promising approach to enhance targeting specificity. For instance, acid-responsive or tumor-associated protease-activated CPPs (ACPPs) can achieve selective activation within tumor tissues, potentially reducing the off-target toxicity associated with conventional CPPs. Third, multifunctional peptide platforms that integrate targeting, delivery, and therapeutic functions may overcome the limitations of single-mechanism approaches. Combining peptides with nanocarriers, immunotherapy, chemotherapy, or radiotherapy could generate synergistic antitumor effects, improving outcomes for heterogeneous gastrointestinal tumors. Finally, incorporating tumor biomarkers and molecular stratification into clinical trial designs is crucial for identifying patient populations most likely to benefit from peptide therapies, thereby enhancing the likelihood of successful clinical translation. Collectively, these strategies suggest that future peptide therapeutics may evolve into multifunctional, precision-guided systems capable of overcoming the complex biological barriers unique to gastrointestinal malignancies.

## 6. Conclusions

In summary, peptide therapeutics occupy a pivotal intersection of molecular biology, peptide chemistry, and oncology, providing a highly versatile and efficient platform for developing next-generation cancer therapies. While challenges remain in in vivo delivery, clinical translation, and manufacturing, rapid advancements in these areas indicate a promising future. Continued interdisciplinary collaboration remains crucial for bridging the gap between laboratory discoveries and clinical application, ultimately realizing the transformative potential of peptide therapeutics in treating gastrointestinal malignancies and improving patient outcomes.

## Figures and Tables

**Table 1:** Roles of peptides in gastrointestinal tumors.

Peptide Name	Peptide Sequence	Function	Type	Tumor
TAT-Inh	YGRKKRRQRRRIVVDSRGFQKL	Inhibits Rac1 activity and suppresses EMT and metastasis by blocking NSD2/Tiam1 [61,62].	Blocking peptides	CRC
Lunasin	SKWQHQQDSCRKQKQGVNLTPCEKHMEKIQGRGDDDDDDDD	Inhibit cancer cell proliferation and transformation by competitively binding to HATs [67,68].	Blocking peptides	CRC
YIGSR	YIGSR	Targeted delivery to 67LR, combined with GEM to overcome resistance [73,74,75].	Delivery peptides	CRC
G17DT	GPWLEEEEE	Induces anti-gastrin antibody production, blocks CCK2-R signaling, inhibits cell proliferation, and induces apoptosis [155,222].	Mimetic peptides	CRC/GC
0020OCV-C02	NSQPVWLCL(RNF43-721) KLRQEVKQNL(TOMM34-299)	For mutant RNF43 [223], combination with UFT/LV therapy improves recurrence prognosis [80,81,82,83].	Mimetic peptides	CRC
Survivin-2B	AYACNTSTL	HLA-A24 restrictive epitope peptide vaccine targeting invivor-2B can promote cell apoptosis [87].	Mimetic peptides	CRC
GX1 Peptide	CGNSNPKSC(cyclic)	GX1-PTX-NLCs deliver PTX specifically to tumor vasculature, inhibiting angiogenesis and exerting antitumor effects [106,107].	Blocking peptides	GC
H-P-6	PQPKVLDS	Prevents *H. pylori*-induced proliferation by inhibiting EGFR signaling [100].	Blocking peptides	GC
GMBP1	ETAPLSTMLSPY	Reverse drug resistance and enhance chemotherapy sensitivity by binding to GRP78 [101,102].	Blocking peptides	GC
TCP-1/TNF-α	CTPSPFSHC	Targeted delivery of TNFα to the GC vascular system, combined with chemotherapy to inhibit tumor growth [108].	Delivery peptides	GC
GEBP11	CTKNSYLMC	Targets vascular imaging to guide surgical resection [109,110,111,112,113].	Delivery peptides	GC
VEGFR1-1084 VEGFR2-169	SYGVLLWEI/RFVPDGNRI	Induce CTL response and inhibit angiogenesis [116].	Mimetic peptides	GC
WW Peptide	WWRRWWRR/(WWRR)n	Inhibits cell migration and metastasis by targeting IQGAP1-ERK complex [224,225].	Blocking peptides	PDAC
CP-FaP2/ CP-FaP3	YGRKKRRQRRR-LELQLRLQELQKQL/ YGRKKRRQRRR-QELQKQLLELQLRL	Disrupt FAM83A-β-catenin interaction, and inhibit Rac1 signaling and EMT [129].	Blocking peptides	PDAC
SKP5	RADVWSFGILLYEMV	Inhibit cell proliferation by blocking the Wnt signaling pathway [130,131].	Blocking peptides	PDAC
GE11	YHWYGYTPQNVI	Specifically binds to EGFR, acting as a delivery ligand to enhance the targeting precision of chemotherapeutic drugs [135].	Mimetic peptides	PDAC
PEP-010	VKKKKIKAEIKIYVETLDDIFEQWAHSEDL	Induces apoptosis by blocking caspase-9/PP2A interaction and releasing caspase-9 [132].	Blocking peptides	PDAC
iRGD	CRGDKGPDC	Opens drug channels and enhances tissue permeability of nanomedicines by targeting vascular integrin αvβ3/β5 [136,137,138,139,140,141].	Delivery peptides	PDAC
LyP-1	CGNSNPKSC	Precise imaging and drug delivery by targeting p32 receptor [144,145,146].	Delivery peptides	PDAC
PTP-PEG	KTLLPTP	Plectin-1-targeted delivery system enhances intracellular drug accumulation and therapeutic efficacy [148,149,150].	Delivery peptides	PDAC
WT1-235/ WT1-126	CMTWNQMNL/ RMFPNAPYL	Induce WT1-specific immune responses and reduce cell viability [159,160,161,162].	Mimetic peptides	PDAC
C7	CTPQTRPNC	Inhibits invasion and metastasis and reduces angiogenesis activity by competitively blocking HGF/c-Met [180].	Blocking peptides	HCC
FFW	RRKFAKFQWI	Induces apoptosis, inhibits migration, and activates tumor suppressor genes by blocking SALL4/NuRD [179].	Blocking peptides	HCC
HM-3	IVRDRAAVPGGGRGD	Inhibits angiogenesis and metastasis and prolongs circulation by blocking αvβ3 integrin [181].	Mimetic peptides	HCC
P58	YLFAIYSFSSL	Inhibits cell proliferation, migration, and invasion, and induces apoptosis by blocking CDP/SOX2 [187].	Blocking peptides	ESCC
DOX-IEK	IEIIIK	Serves as a drug delivery platform that facilitates pH-sensitive targeted release [189].	Delivery peptides	ESCC

## Data Availability

Not applicable.
